# Delay in development and behavioural abnormalities in the absence of p53 in zebrafish

**DOI:** 10.1371/journal.pone.0220069

**Published:** 2019-07-19

**Authors:** Seham Elabd, Nuzhat Amna Jabeen, Vanessa Gerber, Ravindra Peravali, Jean-Christoph Bourdon, Shilpa Kancherla, Daniela Vallone, Christine Blattner

**Affiliations:** 1 Karlsruhe Institute of Technology, Institute of Toxicology and Genetics, Eggenstein-Leopoldshafen, Germany; 2 Human Physiology Department, Medical Research Institute, Alexandria University, Hadara, Alexandria, Egypt; 3 Dundee Cancer Centre, University of Dundee, Ninewells Hospital and Medical School, Dundee, United Kingdom; University of California Davis, UNITED STATES

## Abstract

p53 is well-known for its tumour-suppressive activity. However, in the past decade it became clear that p53 is also involved in other processes including stem cell proliferation, differentiation and animal development. To investigate the role of p53 in early embryonic development, we targeted p53 by CRISPR/Cas9 to make a p53 knock-out zebrafish (*Danio rerio*). Our data show developmental and behavioural effects in p53-deficient zebrafish embryos and larvae. Specifically, we found that early development of zebrafish was clearly delayed in the absence of p53. However, after 1 day (1 dpf), the p53-deficient embryos appeared to recover, as evidenced by a similar level of pigmentation at 26 hpf, similar size of the eye at 4 dpf and only a minor difference in body size at 4 dpf compared to p53 wild-type siblings. The recovery of development after 1 dpf in p53-deficient embryos could be due to a compensatory mechanism involving other p53 family members. p63 and p73 were found over-expressed with respect to wild-type siblings. However, despite this adaptation, the hatching time remained delayed in p53^-/-^ zebrafish. In addition to differences in development, p53-null zebrafish embryos also showed differences in behaviour. We observed an overall reduced activity and a reduced travel distance under non-stressed conditions and after exposing the larvae to vibration. We also observed a longer latency until the larvae started to move after touching with a needle. Overall, these data indicate that p53 is involved in early development and locomotion activities.

## Introduction

Since their establishment as a model animal, zebrafish (*Danio rerio*) have been used in basic research and disease modelling. Because the embryos develop outside of the body of the mother and are optically clear, zebrafish became the preferred model system for studying the progression of development.

p53 is a protein that is most well-known for its tumour-suppressive activities. In response to DNA damage and some other forms of cellular stress, the p53 protein is stabilised and post-translationally modified, resulting in high levels of active p53 [1]. Functionally, p53 is a transcription factor that binds to promoters of target genes leading to their transcriptional activation. p53 primarily regulates genes that control the cell cycle or induce apoptosis [2]. p53 can also induce cell death in a transcription-independent manner, by interacting with pro- and anti-apoptotic proteins in the cytoplasm [3]. p53 is a member of a larger protein family that also includes p63 and p73 as well as different splice versions of these three proteins [4]. p53, p63 and p73 are derived from a common ancestor and show a high homology, particularly in the DNA binding region [5]. Although all three genes have different activities and regulate transcription of unique target genes, they also regulate some common target genes under certain conditions.

The p53 gene is structurally and functionally conserved in zebrafish and ubiquitously expressed in the early embryo [6,7]. Like in mice and humans, p53 acts as a tumour-suppressor protein in zebrafish and defects caused by reductions in Mdm2-levels in zebrafish are rescued by knocking-down p53 [8–10], showing the conservation of its regulation and ensuring transferability of data to higher vertebrates. For investigating p53 regulation and function in zebrafish in the past it was necessary to use a p53-mutant fish line or p53 antisense-morpholino-oligonucleotides due to the unavailability of a p53^-/-^ zebrafish line. These approaches had, however, severe drawbacks. Unlike other tumour-suppressor genes, that become inactive after mutation, p53 can turn into an oncogene when it is mutated and the injection of morpholino-oligonucleotides frequently results in a characteristic induction of neural death [11, 12].

More recent investigations showed that the activity of p53 is not restricted to the DNA damage response or to the induction of cell cycle arrest and cell death. p53 has, moreover, also been shown to regulate cellular metabolism, autophagy and stem cell proliferation and differentiation [13–15]. However, not much is known about the activities of p53 during development or for behaviour.

In order to investigate the role of p53 during early development and for behaviour, we generated a p53-knock-out zebrafish line using the CRISPR/Cas9 system. By targeting exon3, a small deletion was introduced into the p53 gene followed by a frameshift resulting in the complete loss of full-length p53 function. We established a homozygous p53^-/-^ zebrafish line and followed the progression of development and the behaviour of zebrafish by microscopy.

## Material and methods

### Generation of p53-knock-out zebrafish

p53 knock-out mutants were generated using CRISPR/Cas9 genome editing [16]. Eggs of the zebrafish AB wild-type strain (European Zebrafish Resource Centre, Karlsruhe) were collected shortly after fertilisation and 2–3 nl of a mixture of a guide-RNA targeting exon 3 of p53 (80 ng/μl f.c), Cas9 protein (100 ng/μl; Invitrogen) and diluted phenol red were injected into the yolk of zebrafish embryos in the one-cell stage using a gas-driven microinjector system (Tritech) and a SMZ645 stereomicroscope (Nikon). A G1 founder with a 10 bp deletion in the third exon of the p53 gene was identified by fin-clipping and sequencing of a PCR product amplified from the target region of the guide RNA. The 10 bp deletion results in a frameshift causing a complete loss of p53 function. The founder was crossed with a wild-type fish of the AB line (F1). Heterozygote animals were identified by fin-clipping and sequencing of a PCR product amplified from the target region of the guide RNA. Heterozygous fish were crossed and homozygous wild-type and p53-knock-out fish (F2) were identified by fin-clipping and sequencing of a PCR product amplified from the target region of the guide RNA. Homozygous wild-type and p53^-/-^ fish were in-crossed to expand the wild-type and p53^-/-^ colonies (F3). The F3 and F4 generations were in-crossed and eggs and embryos (F4, F5) were used for experiments.

### Zebrafish maintenance and embryo handling

Zebrafish of the AB wild-type strain and of the p53-knock-out line were maintained according to standard procedures. The animals were kept at 28°C +/- 1 on a 14 h light and 10 h dark cycle and fed twice a day with dry food and once a day with live artemia. The system water was sterilised by UV-irradiation. Health was monitored by daily inspection. Sentinel fish were sent for microbiological analysis every 6 months. For breeding, male and female adult fish were separated in the evening. The next morning, the separated fish were allowed to mate in standard breeding tanks.

For ionising irradiation, fertilised eggs or embryos were collected and incubated in egg water (60 μg/ml f.c. sea salts, Instant Ocean Spectrum Brands, Blacksburg, VA) at 28°C +/- 1. The eggs/embryos were irradiated at a dose rate of 14 Gray/h. Thereafter, eggs/embryos were transferred back to 28°C +/- 1 and further incubated until they were analysed.

For monitoring development, zebrafish eggs were transferred to round bottom 96-well plates and placed under an automated microscope. Every hour a picture was taken of each embryo. The water (60 μg/ml f.c. sea salts, Instant Ocean Spectrum Brands, Blacksburg, VA) was exchanged once a day.

All animal experiments were performed at least twice.

### Cell culture

Cell lines were established from wild-type and p53^-/-^ zebrafish embryos as described previously [17]. Established zebrafish cell lines were cultured in Leibvitz’s L-15 medium (GIBCO) supplemented with 15% FBS, 1% penicillin/streptomycin and 0.1% Gentamycin at 28°C. UV-irradiation was performed with a germicidal UVC lamp (254 nm) at a dose of 30 J/m2. Prior to irradiation, the culture medium was removed from the cells and kept sterile. The cells were washed with PBS and exposed to UVC light until the desired dose was reached, or mock treated for control. Thereafter, the culture medium was added back to the cells and the cells were incubated for 16 hours.

### Cell lysis and Western blotting

Zebrafish embryos were homogenised in RIPA-buffer (50 mM Tris pH7.5, 150 mM NaCl, 1% NP40, 0.5% Na-deoxycholate, 5mM PMSF) with a pestil in a reaction tube. Zebrafish cells were lysed as described earlier [18]. Western blotting was performed as described [18].

Anti-zebrafish p53 (GTX12813, Genetex, Irvine, California, USA) and anti-Vinculin (V9131, Sigma-Aldrich, St. Louis, Missouri, USA) antibodies were used as primary antibodies. HRP-coupled anti-mouse and anti-rabbit antibodies (Dako, Glostrup, Denmark) were used for secondary antibodies

### q-RT-PCs

30–50 fertilised eggs or 10 to 30 embryos were lysed in PeqGold Trifast (Peqlab, Erlangen, Germany). RNA was prepared according to the manufacturer’s recommendation. q-RT-PCR was performed as described [18]. Primer sequences are shown in S1 Table.

### Vibration, touch and activity assays

Three behavioural phenotypes were investigated: the escape reaction after touch and after vibration, and the overall locomotion activity. For all the experiments, the larvae were first acclimatisfered in the experimental environment for 30 minutes to remove potential stress factors. For the “touch experiment”, larvae at 3 dpf were placed in a petri-dish. The larvae where then touched with a needle at the tail region. A high speed camera (1000 frames per second) was recording from above the petri dish. For the response to vibration, larvae at 4 and 5 dpf were placed in a petri-dish and subjected to a 500 hertz vibratory pulse that lasted for 2 milliseconds. A high speed camera (1000 frames per second) was recording from above the petri dish. For monitoring spontaneous locomotion, larvae were placed into petri-dishes in a custom constructed robotic imaging chamber. The petri-dishes were placed in a dark-field set up and the locomotion was monitored using a camera operating at 20 frames per second. In all the experiments, the response to stimuli or locomotion was analysed using custom developed software.

### Ethics-statement

The research was carried out according to the German law and approved by the Regierungspraesidium Karlsruhe under approval number 35–9185.81/G-133/17.

### Statistics

Statistical comparisons between two groups were made using student’s t-test. Statistical significance was set to p < 0.05.

## Results

### Generation of a zebrafish p53^-/-^ line

**Fig 1:**
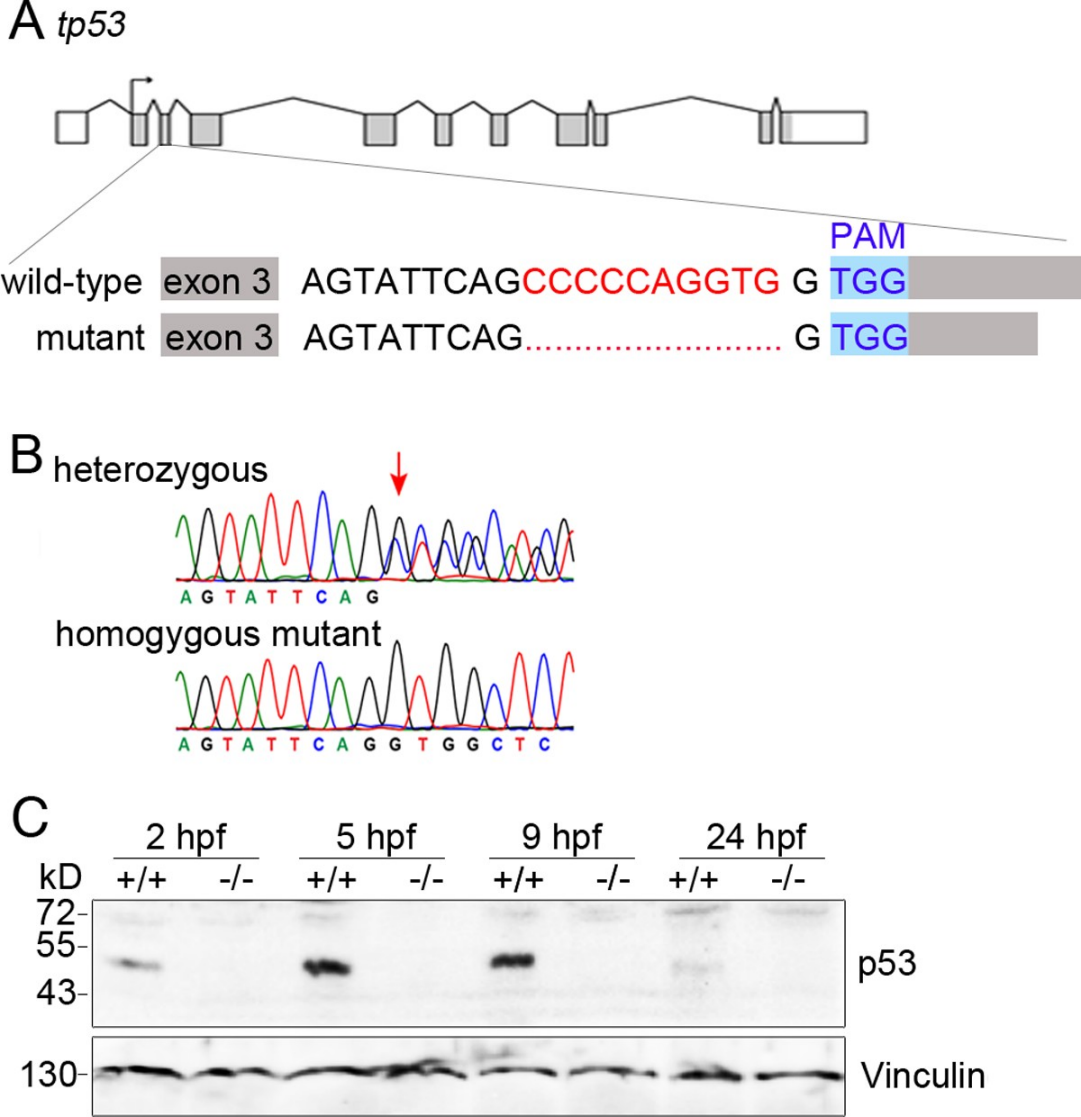
Generation of p53-KO zebrafish. **(A)** Schematic drawing of the CRISPR/Cas9 approach to target zebrafish p53. **(B)** Sequencing data for p53^+/-^ (heterozygous) and p53^-/-^ (homozygous mutant) zebrafisch. **(C)** +/+ (wild-type) and -/- (p53^-/-^) zebrafish embryos were lysed at the indicated times post fertilisation and the p53 protein was monitored by Western blotting. Immunodetection of Vinculin was performed for loading control.

For knocking-out full-length p53 in zebrafish, we applied the CRISPR/Cas9 system. This system is based on the generation of DNA double strand breaks, which are introduced by the Cas9 protein after being directed to a particular sequence in the target gene via a guide RNA [19]. The double strand breaks are most frequently rejoined by NHEJ (non-homologous endjoining), a DNA repair pathway that usually results in small indels at the site of the lesion. We designed a guide RNA targeting exon 3 of p53 (Fig 1A) and microinjected this guide RNA together with Cas9 and traces of phenol red, to mark injected cells, into fertilised one-cell-stage zebrafish eggs. We monitored the efficiency of the guide RNA and the CRISPR/Cas9 system by fin-clipping followed by genomic PCR and sequencing and observed an almost 100% targeting efficiency. Zebrafish with a 10-base pair deletion in exon 3 of the p53 gene followed by a frameshift were selected for further processing (Fig 1A and 1B). This mutation abolishes the expression of full-length p53 protein. Zebrafish with the targeted *p53* gene were raised to sexual maturity and out-crossed with wild-type zebrafish. The progeny (F1) were screened by PCR for heterozygosity in the p53 locus. Heterozygous F1 zebrafish were in-crossed and the progeny (F2) were assessed for the mutation in the *p53* gene by genomic PCR and sequencing. Wild-type and homozygous p53-knock-out animals were each in-crossed (F3) to expand the colonies. Fertilised eggs and embryos of the F3 generation were assessed for the absence of p53 by genomic PCR and sequencing and by Western blotting, and were, together with an F4 generation from a further in-cross, used for experiments. Consistent with earlier reports [6,20], we observed high levels of p53 from 2 hpf (hours post fertilisation) to 9 hpf in wild-type zebrafish. At 24 hpf, p53 was barely detectable. In p53-/- fish, p53 was not detectable at all analysed time points (Fig 1C).

### DNA damage response in p53^-/-^ zebrafish

p53 is well-known for its promoting-cell death activity in response to DNA damage. After DNA damage, p53 is stabilised, resulting in a strong increase in its amount, and post-translationally modified. Both alterations together lead to a strong activation of the transcription of p53 target genes and result in cell cycle arrest and cell death [1]. This p53-dependent DNA damage response is also observed in zebrafish where ionising radiation leads to a strong increase in the mortality of zebrafish that is reduced after inactivation of p53 [21].

**Fig 2:**
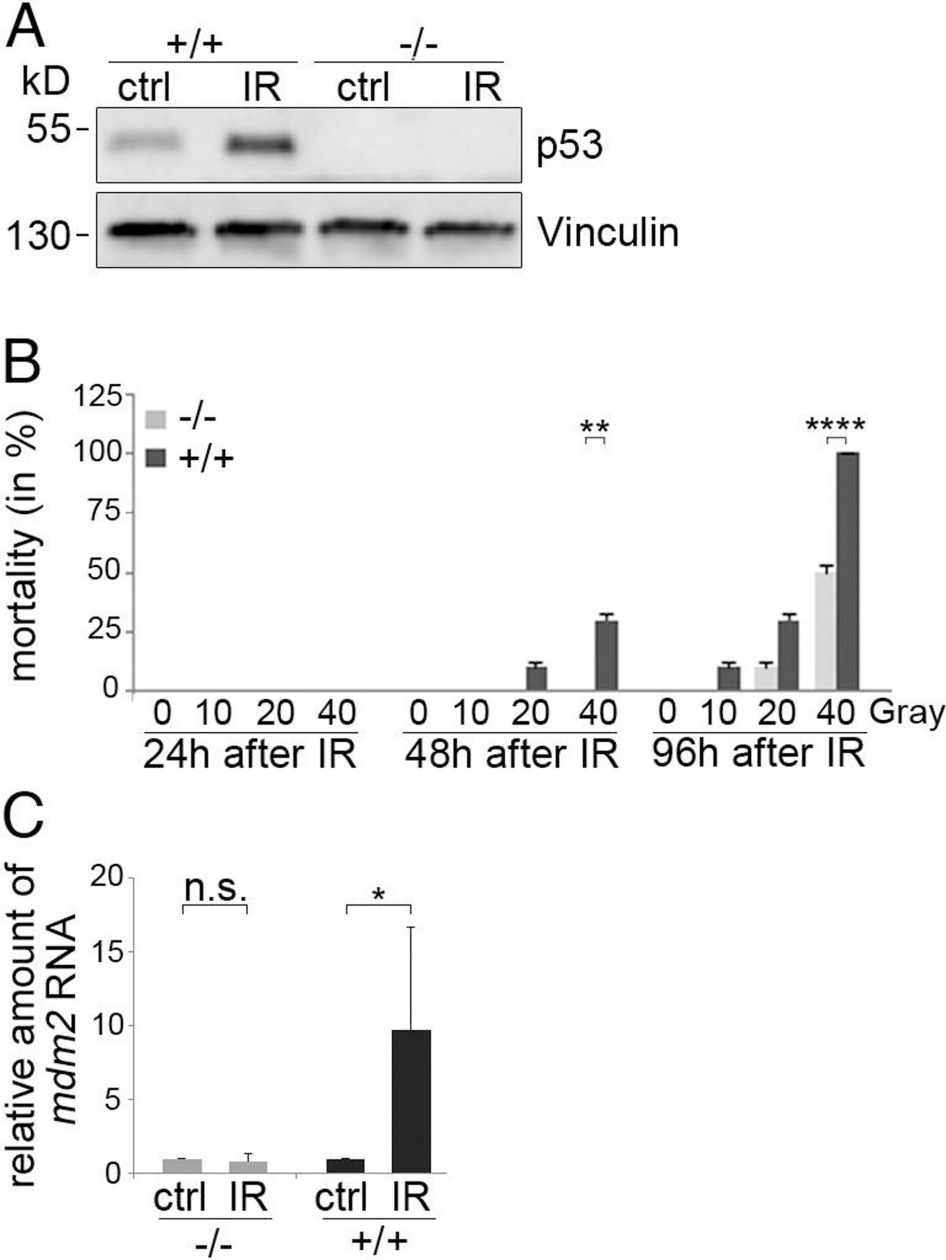
Decreased mortality after ionising irradiation of p53^-/-^ zebrafish embryos. **(A)** wild-type (+/+) and p53^-/-^ (-/-) zebrafish embryos were irradiated with 6.5 Gray at 5 hpf and harvested at 9 hpf. The zebrafish embryos were lysed and p53 levels were monitored by Western blotting. Immunodetection of Vinculin was performed for loading control. **(B)** Wildtype (+/+) and p53^-/-^ (-/-) zebrafish embryos were irradiated at 24 hpf with the indicated doses of ionising radiation. Alive and dead embryos were counted at 1, 2 and 4 days after irradiation. The percentage of dead embryos was calculated and plotted. **: p < 0.01; ****: p < 0.001. **(C)** Wild-type (+/+) and p53^-/-^ (-/-) zebrafish embryos were irradiated with 12 Gray at 6 hpf and harvested at 25 hpf. RNA was prepared and the amounts of *mdm2* and *gapdh* RNA were determined by qRT-PCR. The relative amount of *mdm2* RNA was calculated by the 2^ delta-CT method. The graph shows mean values and standard deviations of 4 (-/-) and 6 (+/+) independent crossings. The relative amount of *mdm2* RNA of mock-treated wild-type cells was set to 1. *: p < 0.05; n.s.: not significant.

To further confirm the functional inactivation of p53 in the p53^-/-^ zebrafish line, we irradiated wild-type and p53^-/-^ zebrafish eggs (F4) at 5 hpf with 6.5 Gray of ionising radiation, incubated the eggs for 4 further hours and monitored p53 abundance by Western blotting. In wild-type animals we observed a strong induction of p53 while we were unable to detect a signal for p53 in the p53^-/-^ siblings (Fig 2A).

Due to the absence of p53, p53^-/-^ zebrafish should be less sensitive to ionising radiation [21]. To test this prediction, we irradiated WT and p53^-/-^ zebrafish embryos at 24 hpf with increasing doses of ionising radiation and monitored their survival. At 24 hours after irradiation (2 dpf), all animals were still alive. At 48 hours after irradiation, wild-type embryos died in a dose dependent manner (Fig 2B) while the p53^-/-^ siblings were still all alive. At 96 hours after irradiation, all wild-type embryos that had received 40 Gray of irradiation were dead while 50% of the p53^-/-^ siblings were still alive. After irradiation with 20 Gray, we also observed a higher mortality in wild-type embryos than in the p53^-/-^ siblings group at 96 hours after irradiation. Irradiation with 10 Gray only killed wild-type embryos at 96 hours after irradiation while all the p53^-/-^ siblings remained alive (Fig 2B). For monitoring p53 activity also at the molecular level, we irradiated fertilised eggs of wild-type and p53^-/-^ zebrafish with ionising radiation and monitored RNA levels of the p53 target gene *mdm2*. We observed a significant increase in the amount of *mdm2* RNA in embryos from wild-type zebrafish but not in the embryos from p53^-/-^ animals confirming that the p53-mediated transcription of the *mdm2* gene is abolished in p53^-/-^ zebrafish (Fig 2C).

We established cell lines from wild-type and p53^-/-^ zebrafish embryos and confirmed the absence of p53 in the p53^-/-^ cell line by Western blotting (S1A Fig). We irradiated wild-type and p53^-/-^ cells with 30 J/m^2^ UVC-light, harvested the cells 16 hours after irradiation and monitored RNA levels of the p53 target genes *mdm2* and *bax*. While the *mdm2* and *bax* mRNAs were strongly induced in the wild-type cells after UVC-irradiation we did not see an increase in these mRNAs in cells from p53^-/-^ zebrafish (S1B Fig).

In summary, these results show that the p53-induced cell death in response to DNA damage is strongly reduced in p53^-/-^ zebrafish, confirming that full-length p53 activity is abolished in this line.

### Delayed early development in zebrafish embryos lacking p53

**Fig 3:**
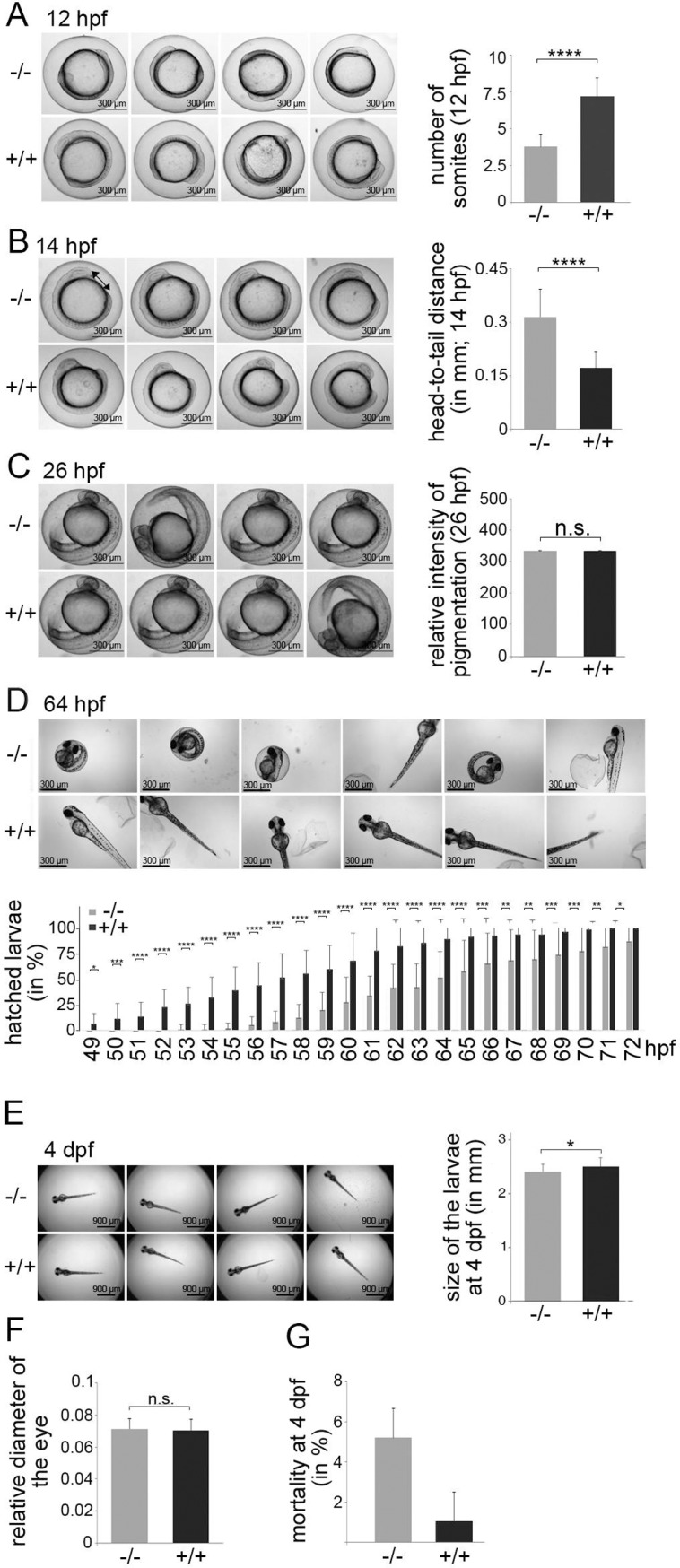
Early development is delayed in p53^-/-^ zebrafish embryos. Wild-type and p53^-/-^ zebrafish were crossed. Fertilised eggs were transferred into a 96 well plate and monitored by automated microscopy. Images were taken every hour. **(A)** Wild-type (+/+) and p53^-/-^ (-/-) zebrafish embryos at 12 hpf. The graph shows mean values and standard deviations of somite numbers of wild-type (n = 50) and p53^-/-^ embryos (n = 42) at 12 hpf. ****: p < 0.001. (**B)** Wild-type (+/+) and p53^-/-^ (-/-) zebrafish embryos at 14 hpf. The graph shows mean values and standard deviations of the distance between the head and the tail of wild-type (n = 48) and p53^-/-^ animals (n = 47). ****: p < 0.001. The arrow shows to the area between the head and the tail that has been measured. **(C)** Wild-type (+/+) and p53^-/-^ (-/-) zebrafish embryos at 26 hpf. The graph shows mean values and standard deviations of the pigmentation intensity of wild-type (n = 13) and p53^-/-^ embryos (n = 14). n.s.: not significant. **(D)** Wild-type (+/+) and p53^-/-^ (-/-) zebrafish embryos at 64 hpf. The graph shows mean values and standard deviations of the numbers of hatched wild-type and p53^-/-^ larvae at the indicated time points. *: p < 0.05; **: p < 0.01; ***: p < 0.005; ****: p < 0.001. **(E)** Wild-type (+/+) and p53^-/-^ (-/-) zebrafish larvae at 4 dpf. The length of the zebrafish embryos was measured at 4 dpf. The graph shows mean values and standard deviations of wild-type (n = 71) and p53^-/-^ (n = 55) larvae. *: p < 0.05. **(F)** The diameter of the eyes of 4 day-old wild-type (+/+) and p53^-/-^ (-/-) larvae was measured, divided by the total lengths of the embryo and plotted. The graph shows mean values and standard deviations of wild-type (n = 71) and p53^-/-^ (n = 55) larvae. n.s.: not significant. **(G)** Wild-type and p53^-/-^ zebrafish were mated. 4 days post fertilisation, vitality of the wild type (+/+) and p53^-/-^ (-/-) larvae was monitored by visual inspection. The graph shows mean values and experimental variation of dead larvae of two independent experiments (n = 48 larvae for each condition and each experiment).

After having confirmed that the full-length p53 protein is inactive in the p53^-/-^ zebrafish line, we monitored the early development of zebrafish. Zebrafish embryos are transparent until pigmentation starts at around 24 hpf. This allowed us to study the progression in development of the zebrafish embryos by light microscopy. We transferred fertilised eggs from wild-type and p53^-/-^ zebrafish into a 96 well plate and placed the plate under an automated Olympus IX 81 microscope that was programmed to take every hour a picture of each well. Soon after gastrulation (circa 10 hpf), we saw differences in the development between wild-type and p53^-/-^ animals. We counted the somites of wild-type and p53^-/-^ zebrafish embryos at 12 hpf. While wild-type embryos (n = 50) had between 5 and 9 somites at this time point, the p53^-/-^ siblings (n = 42) showed only between 3 and 5 somites (Fig 3A).

We followed the development further over time and observed that wild-type embryos were significantly larger in size in comparison to age matched p53^-/-^ siblings. When we measured the distance between the head and the tail of the zebrafish embryos at 14 hpf we found that this distance was between 100 μm and 260 nm in the wild-type group (n = 48) and between 150 μm and 570 μm in the p53^-/-^ siblings group (n = 47; Fig 3B).

At around 24 hpf pigmentation of the embryo starts [22]. We wondered whether pigmentation was also delayed in the p53^-/-^ animals and determined the intensity of the pigmentation at 26 hpf. However, we observed no difference in the pigmentation of the wild-type and p53^-/-^ embryos (Fig 3C).

Between 49 and 72 hpf at 28°C the embryos usually leave the chorion [22]. In line with this observation, the first wild-type larvae hatched at 49 hpf and all of the wild-type larvae had hatched at 72 hpf (Fig 3D). This was in strong contrast to the p53^-/-^ siblings where the first larvae hatched at 53 hpf, and by the end of the observation time at 72 hpf some p53^-/-^ larvae were still in the chorion (Fig 3D).

After hatching, we monitored the size of the larvae. Surprisingly, despite the significant difference in size at 14 hpf and the delay in hatching, we observed only an about 5%, reduction in the total lengths of the p53^-/-^ larvae at 4 dpf (days post fertilisation; Fig 3E). When we measured the diameter of the eye at this time point, we also did not find a difference between wild-type animals and p53^-/-^ siblings (Fig 3F).

Of note, although more than 90% of both wild-type and p53^-/-^ embryos survived until the end of the observation time (5 dpf), we observed a clear increase in the number of dead embryos in the p53^-/-^ cohort (Fig 3G).

**Fig 4:**
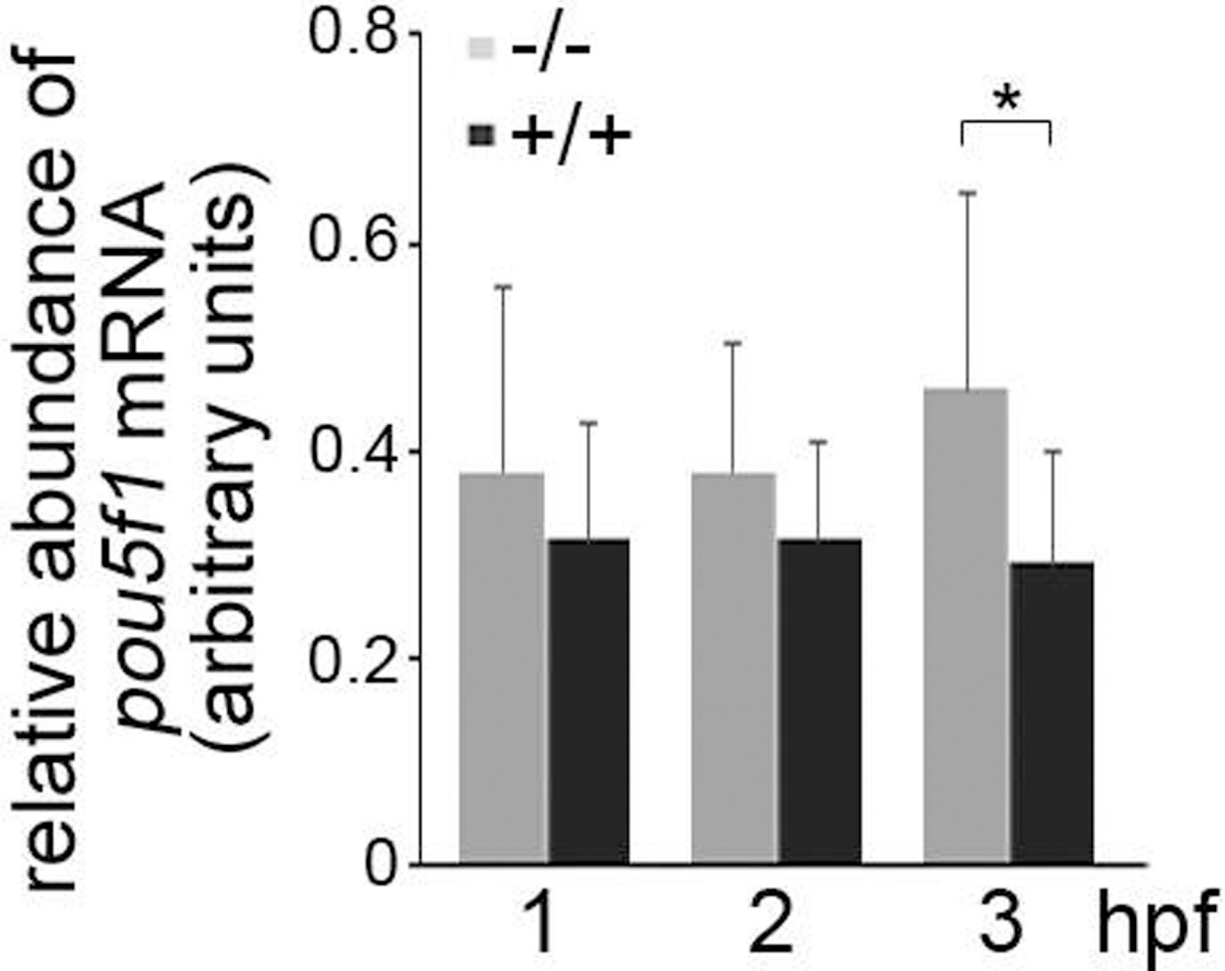
Absence of p53 increases *pou5f1* RNA levels. Wild-type and p53^-/-^ zebrafish were each in-crossed. Fertilised eggs were harvested at 1, 2 and 3 hpf. RNA was prepared and the amounts of *pou5f1* and *gapdh* RNA were determined by qRT-PCR. The relative amount of *pou5f1* RNA was calculated by the 2^ delta-CT method. The graph shows mean values and standard deviations of 6 to 11 independent crossings. *: p < 0.05.

Most interestingly, when murine embryonic stem cells (mESCs) are differentiated with retinoic acid, the amount of the Oct3/4 protein, a member of the POU domain protein family that is critical for the self-renewal of undifferentiated embryonic stem cells, is rapidly decreased [23]. This rapid decrease in Oct3/4 levels is, however, delayed when mESCs with genetically deleted p53 are treated with retinoic acid (S2 Fig). Since we had observed a delay in the development of p53^-/-^ zebrafish embryos, we wondered whether the absence of p53 in the p53-knock-out line also affects expression of the zebrafish orthologue of the *oct3/4* gene. We harvested eggs from wild-type and p53^-/-^ zebrafish during the first 3 hours after fertilisation and monitored expression of the *pou5f1* gene by qRT-PCR. At 1 and 2 hpfs, wild-type and their p53^-/-^ siblings contained about the same amount of *pou5f1* mRNA. Thereafter, at 3 hpf, the expression of *pou5f1* RNA decreased in the wild-type embryos while we observed a further increase in the p53^-/-^ embryos resulting in a significant difference in the amount of *pou5f1* mRNA levels between p53^-/-^ zebrafish embryos and their wild-type siblings (Fig 4). This result suggests that in the absence of p53, high amounts of Oct3/4 delay the differentiation of stem cells.

### Absence of full-length p53 increases expression of p63 and p73

**Fig 5:**
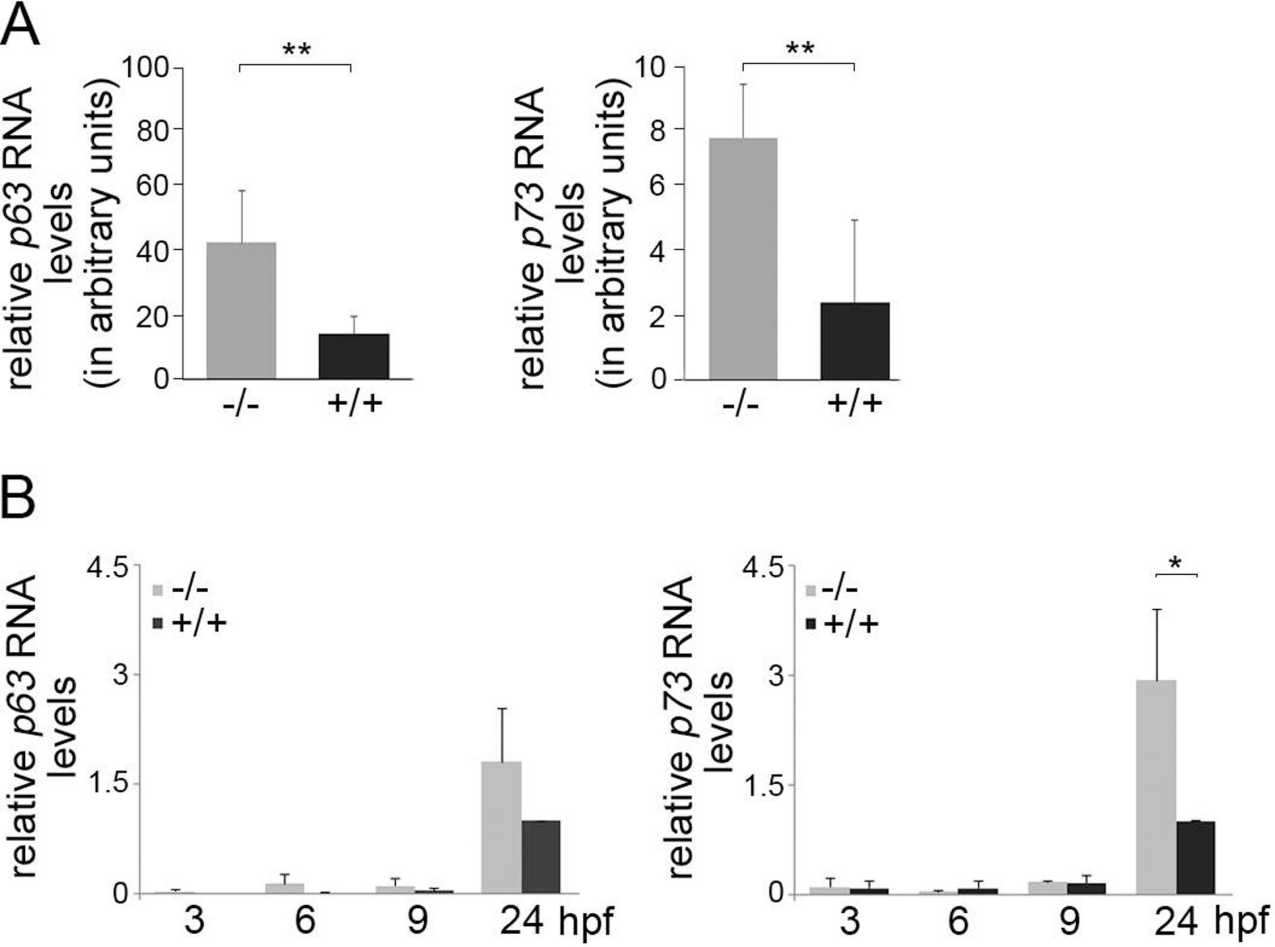
p63 and p73 levels are increased in p53^-/-^zebrafish embryos and larvae. **(A)** Wildtype (+/+) zebrafish larvae (3 dpf) and age matched p53^-/-^ larvae were lysed. RNA was prepared and transcribed into cDNA. *p63*, *p73* and *gapdh* RNA levels were determined by qRT-PCR. Relative levels of *p63* and *p73* RNA were calculated by the 2^ delta-CT method. The graph shows mean values and standard deviations of 4 sets of larvae (2 x 10, 15 and 30 animals each). **: p<0.01. **(B)** Wild-type and p53^-/-^ zebrafish were each in-crossed. Fertilised eggs were harvested at 3, 6, 9 and 24 hpf. RNA was prepared and the levels of *p63*, *p73* and *gapdh* RNA were determined by qRT-PCR. The relative amounts of *p63* and *p73* RNA were calculated by the 2^ delta-CT method. The graph shows mean values and standard deviations of 3 independent experiments. The relative amount of *p63* and *p73* RNA of wild-type cells at 24 hpf was set to 1.

While we observed a strong delay in the development of p53^-/-^ embryos during the first day of development, pigmentation, which takes place at the second day of development, was indistinguishable between wild-type embryos and their p53^-/-^ siblings. Since pigmentation of the embryo is another marker of ongoing development, the absence of a difference in pigmentation intensity suggests that p53^-/-^ embryos have caught-up with development after the first 24 hours. Most interestingly, when we analysed wild-type and p53^-/-^ zebrafish larvae for the presence of the p53 protein, we were unable to detect p53 at 3 dpf (S3A Fig). However, we observed signals at 60 and 70 kD that were stronger in the lysates from p53^-/-^ animals (S3A Fig). Since the antibody that we used for detecting p53 was raised against the DNA binding domain, a domain of high homology between the different p53 family members [24], we speculated that the stronger bands in the lysates from p53^-/-^ larvae could represent p63 and p73. When we monitored the amounts of *p63* and *p73* RNA, these RNAs were also strongly elevated in p53^-/-^ larvae (Fig 5A). We then wondered from which time point during development this increase in p63 and p73 RNA is apparent. Therefore, we harvested fertilised fish eggs at different time points after fertilisation and monitored abundance of *p63* and *p73* RNA by qRT-PCR. The overall amount of *p63* and *p73* RNA was quite low during the first nine hours of development. However, already at the earliest time point of our observation (3 hpf), we observed a 4-fold increase in *p63* RNA in the eggs from p53^-/-^ animals in comparison to their wild-type siblings. This was, however, not the case for *p73*. Here we observed similar levels of RNA in fertilised eggs from wild-type and p53^-/-^ animals (Fig 5B). At 9 hpf, we saw a minor increase also in p73 RNA levels in fertilised eggs from p53^-/-^ zebrafish (Fig 5B). Between 9 hpf and 24 hpf, the overall amount of *p63* and *p73* RNA increased in fertilised eggs from wild-type and p53^-/-^ animals leading to an about 2-fold increase in *p63* RNA and an about 3-fold increase in *p73* RNA in p53^-/-^ embryos in comparison to their wild-type siblings (Fig 5B).

Consistently, when we knocked-down p53 in human HCT116 cells, we also observed an increase in the amount of p63. At the same time, we observed a reduction in the levels of the p53 target genes p21 and Mdm2, confirming the reduction in p53 activity and indicating that the p53 family members p63 and p73 cannot fully compensate for the loss of p53 (S3B Fig).

### p53^-/-^ zebrafish larvae show an altered kinetic behaviour

**Fig 6:**
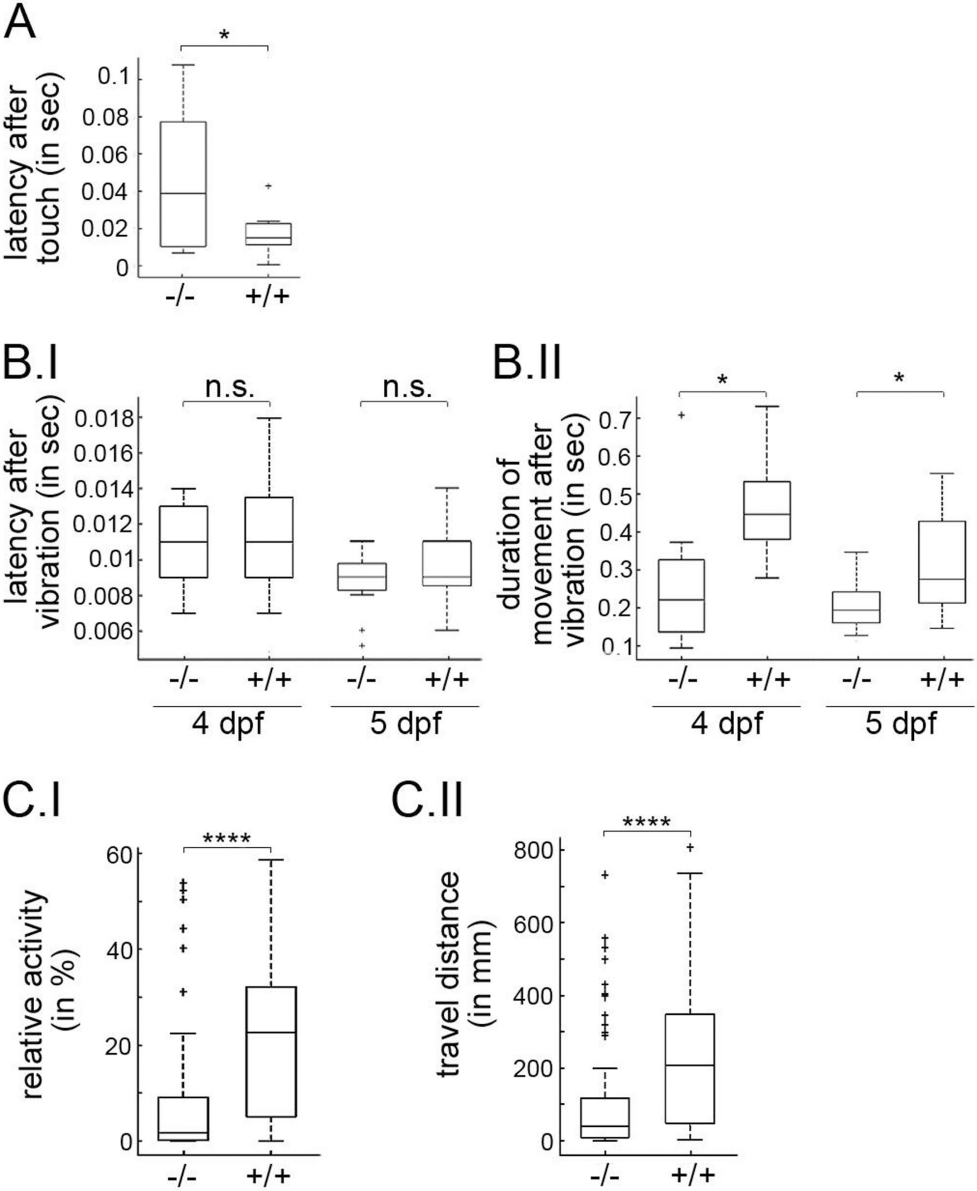
p53^-/-^ zebrafish larvae show an altered response to touch and vibration. **(A)** Wild-type (+/+) and p53^-/-^ (-/-) zebrafish larvae of 4 dpf were allowed to acclimatise to the experimental environment. Then the larvae were touched with a small needle and watched by live-imaging microscopy. The numbers of frames between the touch impulse and the start of movement were counted and the time of latency was calculated. The graph shows mean values and standard deviation of the latency of wild-type (n = 11) and p53^-/-^ (n = 7) larvae. *: p < 0.05. **(B.I)** Wild-type (+/+) and p53^-/-^ (-/-) zebrafish larvae of the indicated age were allowed to acclimatise to the experimental environment. Then the larvae were exposed to vibration and watched by live-imaging microscopy. The numbers of frames between the vibration impulse and the start of movement were counted and the time of latency was calculated. The graph shows mean values and standard deviation of the latency of wild-type and p53^-/-^ larvae at 4 dpf (n = 9) and at 5 dpf (+/+: n = 20; -/-: n = 19). n.s: non significant. **(B.II)** Wild-type (+/+) and p53^-/-^ (-/-) zebrafish larvae of the indicated age were allowed to acclimatise to the experimental environment. Then the larvae were exposed to vibration and watched by live-imaging microscopy until they stopped the movement. The numbers of frames between the start of movement and the end of movement were counted and the duration of movement was calculated. The graph shows mean values and standard deviations of the same number of animals as in part B.I. *: p < 0.05. **(C.I)** The overall activity of the larvae was calculated by measuring the time during which the larvae were moving and the time for which they were idle. The figure shows the percentage of the time for which the larvae were moving over a period of 4 hours (wild-type: n = 54; -/-: n = 60). **(C.II)** The distance travelled by the larvae in 2 minutes averaged over a period of 4 hours was computed. (wild-type: n = 54; -/-: n = 60). ****: p < 0.001.

The role of p53 in the DNA damage response, stem cell proliferation, autophagy or metabolism is well-documented [13,15]. However, less is known about its involvement in behaviour. We used the p53^-/-^ zebrafish line to investigate whether the absence of p53 might also affect the behaviour of zebrafish larvae. Zebrafish larvae are very sensitive to touch and vibration. Soon after these impulses are applied, the larvae respond and demonstrate an escape response. The escape response is governed by two parameters: (i) the latency which is a measure of the time taken by the larvae to respond to the stimulus and (ii) the duration of the response, until the larvae comes to rest. The response to stimuli is very robust and repeatable in wild type zebrafish larvae. We used this read-out to monitor eventual defects in the behaviour of p53^-/-^ zebrafish larvae. At 4 dpf, we first let the larvae acclimatise to the experimental environment. We then touched the larvae with a small needle and measured the time until they started swimming. Wild-type animals started very soon and required only a latency of 0.005 to 0.045 sec until they began swimming. In our experiment, ten out of eleven wild-type larvae had started the escape response before 0.025 sec after touch (Fig 6A). This was in strong contrast to the p53^-/-^ larvae that not only presented a much larger variation of the latency time before they responded to the touching stimulus, they also needed significantly more time after the touch stimulus before they started the escape response (Fig 6A). Interestingly, the situation was different when we exposed the animals to vibration as a second and different stimulus. Here, we did not observe any significant difference in the latency between wild-type and p53^-/-^ larvae (Fig 6B.I). However, we observed a much shorter time of movement for p53^-/-^ larvae both at 4 dpf and at 5 dpf than for their wild-type siblings (Fig 6B.II).

We also investigated the overall locomotion activity of wild-type and p53^-/-^ zebrafish larvae. This activity was monitored using a robotic system carrying a camera that was placed above a petri dish in which the embryos were freely swimming around. The larvae were placed in a dark field set up and their locomotor activity was monitored for several hours. Overall, we observed a significantly reduced locomotor activity of p53^-/-^ animals (Fig 6C.I). Also the distance that the p53^-/-^ larvae travelled was much smaller than that of the wild-type siblings (Fig 6C.II). These results indicate that p53 is also required for kinetic activities and for a proper physiologic response to perturbations in the environment.

## Discussion

### Generation of a p53^-/-^ zebrafish line and the response to DNA damage

We created a zebrafish line with a true inactivation of the p53 tumour suppressor protein. Such a line was not available in the past, which made it necessary to use morpholino-oligonucleotides to knock-down p53 or a mutant fish line for investigating p53 function in zebrafish. Both strategies were, however, only second choice since the ENU-p53 mutant has a mutation in the DNA binding domain and mutations in this region of p53 frequently result in a gain of oncogenic properties [8,11]. Also the use of morpholino-oligonucleotides has side effects such as a frequent induction of neural death in zebrafish embryos [12]. The establishment of cell lines from wild-type and p53^-/-^ zebrafish embryos furthermore allows the investigation of the regulation of p53 in zebrafish without the need for animal experiments. The zebrafish p53^-/-^ cell line showed no induction of *mdm2* or *bax* RNA after UV-irradiation, consistent with the reduction in p53 target gene expression in human and murine p53 cell lines and tissues [25,26].

Exposure of wild-type and p53^-/-^ zebrafish embryos to ionising irradiation resulted in a significantly better survival of the p53^-/-^ embryos compared to their wild-type siblings. This result is in line with an earlier described radioprotection by mutated p53 or after suppression of wild-type p53 with antisense morpholino-oligonucleotides [8,21].

### Developmental delay in p53^-/-^ embryos

When we compared the early development of p53^-/-^ embryos with their wild-type siblings, we observed a significant delay. Both the number of somites and the overall size of the embryo were significantly reduced in the knock-out mutant. The differences in the development were most striking during the first day after fertilisation. Developmental signatures that are apparent at later stages, like pigmentation of the embryo, were not affected. This influence of p53 mostly on the early development is in line with its expression. Consistent with earlier reports [6,20], we observed high p53 levels during the first nine hours after fertilisation. The high expression levels of p53 declined thereafter to undetectable levels. This almost complete disappearance of p53 from the developing embryo indicates that p53 may be of less general importance during later stages of development. Of note, p53 protein levels were high prior to the maternal to zygotic transition of the transcriptome [27] suggesting that the high p53 levels were of maternal origin. At the time when p53 protein levels were decreased, mRNA levels of the p53 family members p63 and p73 were strongly increased. Most interestingly, expression of p63 and p73 was significantly higher in p53^-/-^ embryos than in their wild-type siblings. Since all three family members have a high degree of homology in the DNA binding domain and since all three have, at least to some degree, overlapping target genes [28] it is most likely that the high amounts of p63 and p73 in the developing embryo compensate for the absence of p53 at the end of the first day after fertilisation. This compensation may also be the reason why mice can develop normally in the absence of p53 [29]. The hypothesis that high levels of p63 and p73 may compensate for the absence of p53 in the p53^-/-^ embryo is in line with a redundant role of p53 and p73 in mesodermal development as shown by the Massagué group [30]. A role of p53 in development is furthermore in line with a reported pro-differentiation activity of p53. Spontaneous differentiation of human embryonic stem cells, for instance, is stimulated when p53 abundance is increased by the drug nutlin [31]. One way by which p53 can induce differentiation is by inducing the expression of the microRNA *miR-145* [32]. miR-145 downregulates Oct4 and thus suppresses pluripotency of human embryonic stem cells [33]. In line with these observations, we observed higher levels of Oct3/4 protein in p53^-/-^ murine embryonic stem cells and of *pou5f1* RNA, the zebrafish homologue of *oct3/4*, in fertilised eggs and early embryos from p53^-/-^ zebrafish. However, in contrast to the results by Xu et al., we observed this increase in *pou5f1* RNA while they found that the regulation of Oct4 by miR-145 occurs at the protein level. We were also quite surprised by the increase in *pou5f1* RNA at these early time points as most genes in early embryos are transcribed after the maternal to zygotic transition of the genome that occurs after 3.5 hpf. However, it has been shown that there are clusters of genes that are induced between the 1 cell stage and 3.5 hpf (pre-MBT1 and pre-MBT2 genes) [27]. We speculate that *pou5f1* belongs to one of these groups.

Although it appears as if p63 and p73 can compensate for the loss of p53, there was still a delay in hatching. We also observed more dead embryos/larvae from p53^-/-^ zebrafish demonstrating that the high expression of the p53 family members p63 and p73 can only partially and not fully compensate for the absence of p53. It is, for instance possible, that one or several genes that are required to start or fulfil the hatching process are not available at the same time or in the same quantity in the p53^-/-^ larvae as in the wild-type siblings. Eventually, the binding of the other p53 family members to the promoter of this (or these) genes is not perfect which may result in reduced expression of this gene and thus in a delay of the hatching process.

### Behavioural differences in p53^-/-^ zebrafish larvae

In the last decade it became clear that p53 is not only involved in the DNA damage response, but also in processes such as autophagy, metabolism or the immune response [13–15]. Our observation that p53 is also involved in the kinetic behaviour of zebrafish further expands the repertoire of p53 activities. We observed that the presence of p53 affects the overall locomotion activity as well as the latency and duration of the escape response. Surprisingly, the latency of the escape response after touch was prolonged by p53^-/-^ larvae but not after vibration showing that the behavioural response of zebrafish larvae is stimulus-specific. We observed, however, a decreased duration of the escape response after vibration of p53^-/-^ animals as well as a reduction in the overall locomotion activity. Whether these defects are caused by neuronal defects or problems with the musculature in the p53^-/-^ animals remains to be determined.

## Supporting information

S1 File Supplementary information. (DOCX)

S1 Fig p53 abundance in cell lines established from wild-type and p53^-/-^ zebrafish embryos. **(A)** Cells from cell lines that have been established from wild-type (+/+) and p53-knock-out (-/-) zebrafish embryos were lysed and p53 abundance was monitored by Western blotting. Immunodetection of Vinculin was performed for loading control. **(B)** Wild-type (+/+) and p53-knock-out (-/-) cells were mock-treated (ctrl) or irradiated with UVC light (UV). 16h after irradiation, cells were harvested. RNA was prepared and the amounts of *mdm2*, *bax* and *gapdh* RNA were determined by qRT-PCR. The relative amounts of *mdm2* and *bax* RNA were calculated by the 2-delta-CT method. The graph shows mean values and standard deviations of 3 (*bax* RNA) or 4 (*mdm2* RNA) independent experiments. The relative amounts of *bax* and *mdm2* RNA of mock-treated wild-type cells were set to 100%. *: p < 0.05; n.s.: not significant.
(TIF)

S2 Fig Differentiation is delayed in p53^-/-^ murine embryonic stem cells. Wild-type and p53^-/-^ murine embryonic stem cells were treated with 0.5 μM retinoic acid (RA) and harvested at the indicated time points. 50 μg of protein were separated on a 10% SDS-PAGE gel and blotted. The blots were consecutively hybridised with antibodies directed against p53, Oct 3/4 and GAPDH for loading control.
(TIF)

S3 Fig Increased expression of p63 and p73 in the absence of p53. **(A)** Wild-type (+/+) zebrafish larvae (3 dpf) and age matched zebrafish larvae with deleted p53 (-/-) were lysed in RIPA buffer. 50 μg of protein were separated on an 8% SDS-PAGE gel and blotted. p53 was detected by Western blotting. Immunodetection of Vinculin was performed for loading control. **(B)** HCT116 cells were transfected either with 15 nM siRNA targeting TP53 (p53 siRNA) in exon7 or with 15 nM of a control siRNA (ctrl siRNA) Proteins were extracted 48h after transfection and analysed by SDS-PAGE. Immunodetection of Ku80 was performed for loading control.
(TIF)

S1 Table Sequences of primers. (DOCX)
