# Supplementary material for: Delay in development and behavioural abnormalities in the absence of p53 in zebrafish
Source: PLoS One. 2019 Jul 19;14(7):e0220069. doi: 10.1371/journal.pone.0220069 (PMC6641203; doi:10.1371/journal.pone.0220069)
Supplement: S1 File — (DOCX) [file pone.0220069.s001.docx]

**Supplementary information**

**Materials and Methods**

*Cell culture*

p53^-/-^ murine embryonic stem cells and the corresponding wild-type stem cell line (D3; Solozoboba et al., 2009) were cultured in DMEM medium (GIBCO) supplemented with 15% fetal bovine serum (FBS), 1 x non-essential amino acids, 0.1 mM β-mercaptoethanol, 1% penicillin/streptomycin and 1000 units/ml LIF. Mouse embryonic fibroblasts that had been irradiated with 6.3 Gray served as feeder cells. The cells were maintained in a humidified atmosphere at 37°C and 6% CO_2_. For differentiation, cells were cultured in DMEM medium (GIBCO) supplemented with 10% fetal bovine serum, 1 x non-essential amino acids, 0.1 mM β-mercaptoethanol, 1% penicillin/streptomycin and 0.5 µM all-trans-retinoic acid (St. Louis, Missouri, USA) for up to 7 days in culture dishes coated with 0.1% gelatin.

HCT116 cells were transfected either with 15nM siRNA targeting TP53 in exon7 (p53 siRNA) or with 15nM of a control siRNA (ctrl siRNA) as previously described (Gadea et al., 2016).

*Cell lysis and Western blotting*

Lysis of murine embryonic stem cells and Western Blotting were performed as described earlier (Rodriguez et al., 2018). HCT116 were lysed 48h after transfection and analyzed by SDS-PAGE as previously described (Gadea et al., 2016).

The following antibodies were used: anti-murine p53 (1C12, Cell Signaling Technology, Cambridge, Massachusetts, USA ), anti-human p53 (DO-1, Nekulova et al., 2013), anti-zebrafish p53 (GTX12813, Genetex, Irvine, California, USA), anti-p63 (Tap63-5.1, Nekulova et al., 2013), Oct 3/4 (C-10, Santa Cruz Biotechnology, Dallas, USA); anti-Gapdh (HyTest Turku, Finland), anti-Vinculin (V9131, Sigma-Aldrich, St. Louis, Missouri, USA), anti-p21 (Ab-1, EA10, OP64, Merck, Darmstadt, Germany), anti-Mdm2 (2A10, ab16895, Abcam. Cambridge, UK).

*q-RT-PCR*

10^6^ cells were lysed in PeqGold Trifast (Peqlab, Erlangen, Germany). RNA was prepared according to the manufacturer’s recommendation. q-RT-PCR was performed as described (Rodrigues et al., 2018). Primer sequences provided in table S1.

**References**

Gadea G, Arsic N, Fernandes K, Diot A, Joruiz SM, Abdallah S, et al. [*TP53* drives invasion through expression of its Δ133p53β variant.](https://www.ncbi.nlm.nih.gov/pubmed/27630122) Elife. 2016; 5. pii: e14734.

Nekulova M, Holcakova J, Nenutil R, Stratmann R, Bouchalova P, Müller P, et al. [Characterization of specific p63 and p63-N-terminal isoform antibodies and their application for immunohistochemistry.](https://www.ncbi.nlm.nih.gov/pubmed/23887585) Virchows Arch. 2013; 463: 415-425.

Rodrigues M, Antonucci I, Elabd S, Kancherla S, Marchisio M, Blattner C, et al. p53 is active in human amniotic fluid stem cells. Stem cells and development. 2018; 27: 1507-1517.

Solozobova V, Rolletschek A, Blattner C. Nuclear accumulation and activation of p53 in mouse embryonic stem cells after ionising irradiation. *BMC Cell Biology*. 2009; 10:46.
