# Supplementary figures and images for: Delay in development and behavioural abnormalities in the absence of p53 in zebrafish
Source: PLoS One. 2019 Jul 19;14(7):e0220069. doi: 10.1371/journal.pone.0220069 (PMC6641203; doi:10.1371/journal.pone.0220069)

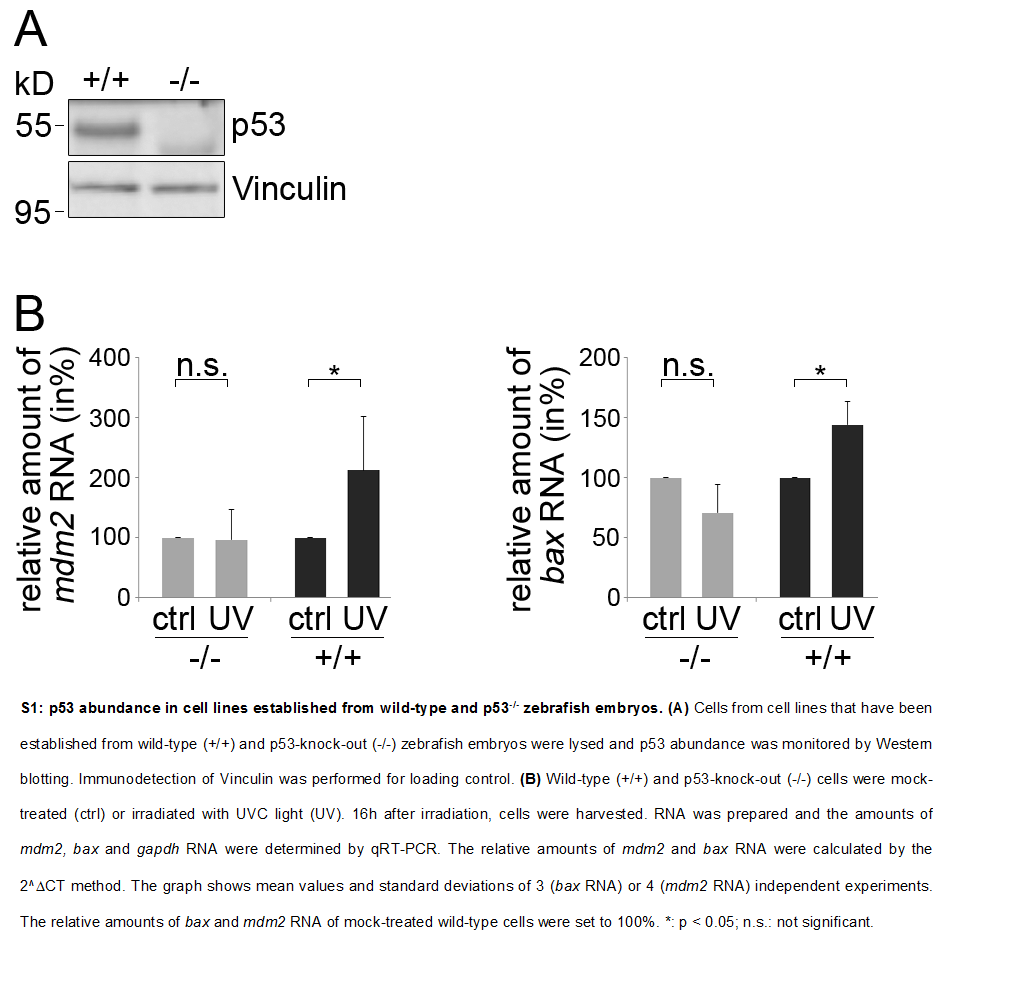

Supplement: S1 Fig — (A) Cells from cell lines that have been established from wild-type (+/+) and p53-knock-out (-/-) zebrafish embryos were lysed and p53 abundance was monitored by Western blotting. Immunodetection of Vinculin was performed for loading control. (B) Wild-type (+/+) and p53-knock-out (-/-) cells were mock-treated (ctrl) or irradiated with UVC light (UV). 16h after irradiation, cells were harvested. RNA was prepared and the amounts of mdm2, bax and gapdh RNA were determined by qRT-PCR. The relative amounts of mdm2 and bax RNA were calculated by the 2-delta-CT method. The graph shows mean values and standard deviations of 3 (bax RNA) or 4 (mdm2 RNA) independent experiments. The relative amounts of bax and mdm2 RNA of mock-treated wild-type cells were set to 100%. *: p < 0.05; n.s.: not significant. (TIF) [file pone.0220069.s002.tif]

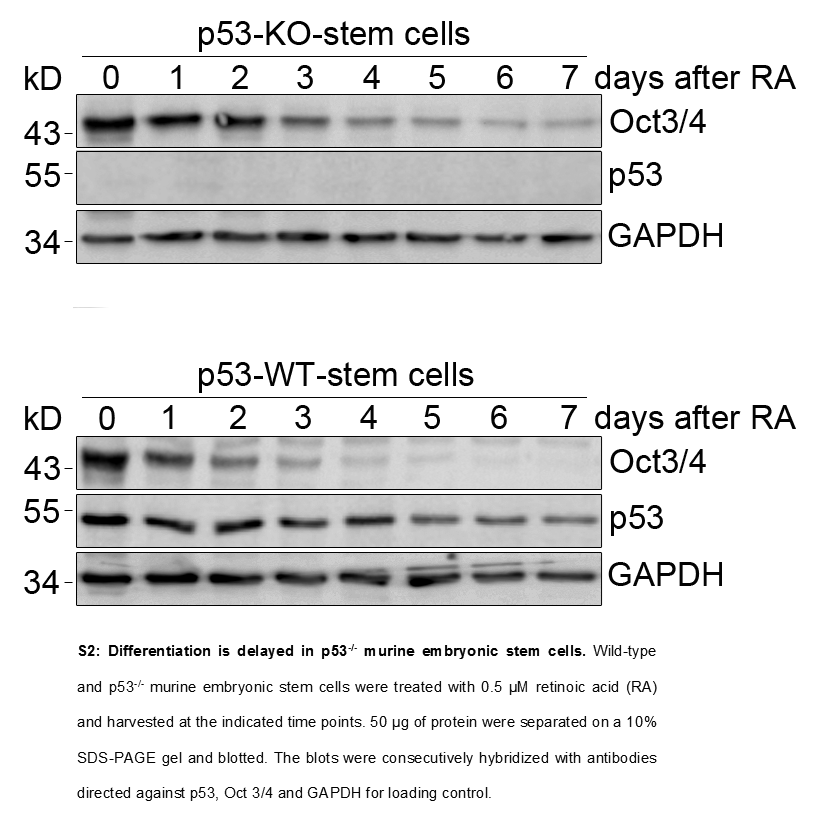

Supplement: S2 Fig — Wild-type and p53-/- murine embryonic stem cells were treated with 0.5 μM retinoic acid (RA) and harvested at the indicated time points. 50 μg of protein were separated on a 10% SDS-PAGE gel and blotted. The blots were consecutively hybridised with antibodies directed against p53, Oct 3/4 and GAPDH for loading control. (TIF) [file pone.0220069.s003.tif]

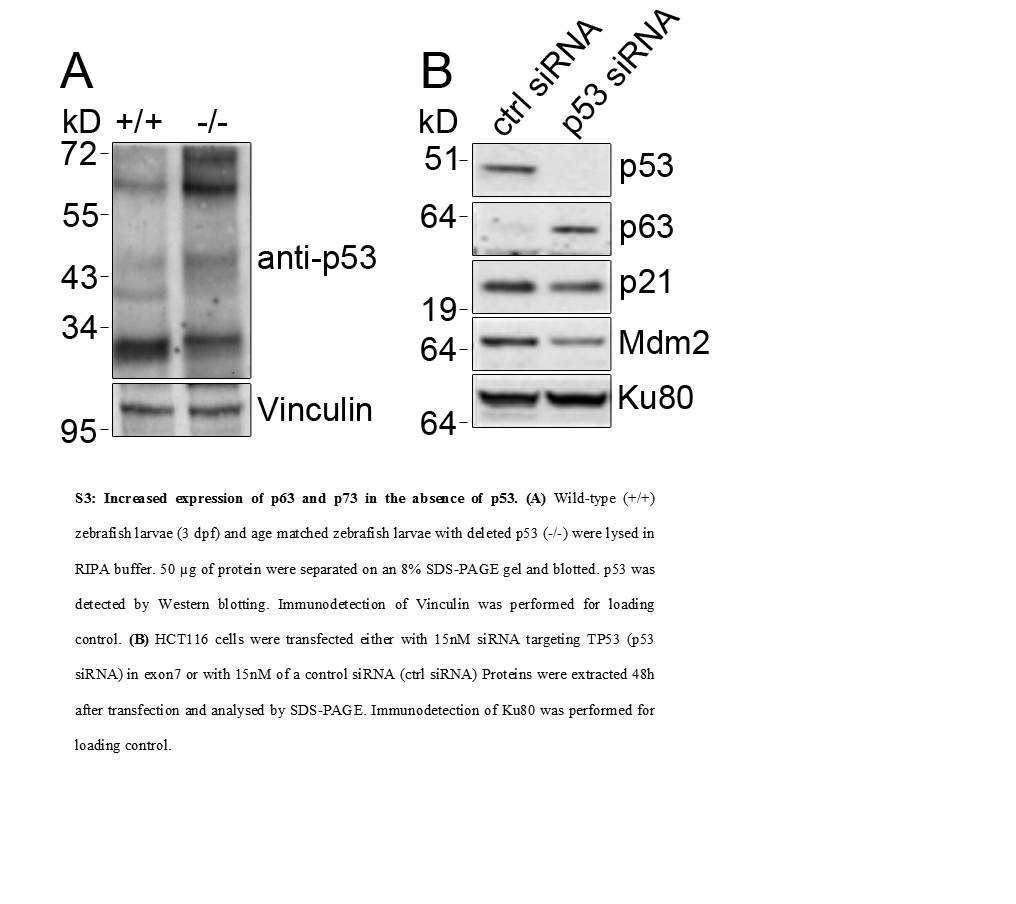

Supplement: S3 Fig — (A) Wild-type (+/+) zebrafish larvae (3 dpf) and age matched zebrafish larvae with deleted p53 (-/-) were lysed in RIPA buffer. 50 μg of protein were separated on an 8% SDS-PAGE gel and blotted. p53 was detected by Western blotting. Immunodetection of Vinculin was performed for loading control. (B) HCT116 cells were transfected either with 15 nM siRNA targeting TP53 (p53 siRNA) in exon7 or with 15 nM of a control siRNA (ctrl siRNA) Proteins were extracted 48h after transfection and analysed by SDS-PAGE. Immunodetection of Ku80 was performed for loading control. (TIF) [file pone.0220069.s004.tif]
