# Supplementary material for: Delay in development and behavioural abnormalities in the absence of p53 in zebrafish
Source: PLoS One. 2019 Jul 19;14(7):e0220069. doi: 10.1371/journal.pone.0220069 (PMC6641203; doi:10.1371/journal.pone.0220069)
Supplement: S1 Table — (DOCX) [file pone.0220069.s005.docx]

| name | Forward primer | Reverse Primer |
| --- | --- | --- |
| p53 | GGG TTG CAG GAG GTG CTT AC | GCA GGC TAG GCT AAG CTA TGA |
| p63 | CCA GCC CCT ACA ACA ACG | AGG ACA CGT CAA AGG TGT |
| p73 | ATC ACA AAC TCG GAC GGG AC | TGG AGA CTC ATA CGG GAC CA |
| GAPDH | TGA GAG CAA TAC CAG CAC CA | GGC AAG CTT ACT GGT ATG GC |
| Mdm2 | TCA GCT GAA GAC GAG TCT AGA GAA C | AGA TAT ACC TAC ATC CGA GTT GCT G |
| Bax | ACA GGG ATG CTG AAG TGA CC | GAA AAG CGC CAC AAC TCT TC |

**Table S1: Sequences of primers**
